# Global Inequities in Precision Medicine and Molecular Cancer Research

**DOI:** 10.3389/fonc.2018.00346

**Published:** 2018-09-04

**Authors:** Thomas M. Drake, Stephen R. Knight, Ewen M. Harrison, Kjetil Søreide

**Affiliations:** ^1^Department of Clinical Surgery, University of Edinburgh, Edinburgh, United Kingdom; ^2^Department of Gastrointestinal Surgery, Stavanger University Hospital, Stavanger, Norway; ^3^Department of Clinical Medicine, University of Bergen, Bergen, Norway

**Keywords:** cancer, surgery, oncology, genomics, low-income, global health, global surgery, precision medicine

## Abstract

Precision medicine based upon molecular testing is heralded as a revolution in how cancer is prevented, diagnosed, and treated. Large efforts across the world aim to conduct comprehensive molecular profiling of disease to inform preclinical models, translational research studies and clinical trials. However, most studies have only been performed in patients from high-income countries. As the burden on non-communicable diseases increases, cancer will become a pressing burden across the world, disproportionately affecting low-middle income settings. There is emerging evidence that the molecular landscape of disease differs geographically and by genetic ancestry, which cannot be explained by environmental factors alone. There is a lack of good quality evidence that characterises the molecular landscape of cancers found in low-middle income countries. As cancer medicine becomes increasingly driven by molecular alterations in high-income settings, low-income settings may become left behind. Further efforts on an international scale must be made by researchers, funders, and policymakers to ensure cancer research addresses disease across the world, so models are not limited to subtypes of disease found in high-income countries. In this review, we discuss differences found in the molecular profiles of tumours worldwide and the implication this has for the future of global cancer care. Finally, we identify several barriers currently limiting progress in this field and innovative solutions, which may address these shortcomings.

## Introduction

The incidence of cancer across the world is increasing, with the number of new cases set to rise by 70% in the next 20 years (1). This rise in incidence is accompanied by a sharp increase in cancer mortality, which disproportionately affects patients in low-middle income countries (2). As the global burden of communicable disease decreases with improvements in prevention, sanitation and treatment, non-communicable diseases such cancer will become a pressing burden. Whilst low and middle-income countries (LMICs) contend with barriers, such as delays in accessing healthcare, advanced disease at presentation, and limited access to treatment; research, and clinical practice in high income countries (HICs) is aimed toward developing treatment strategies tailored to individual patient characteristics and tumour biology.

**Figure 1:**
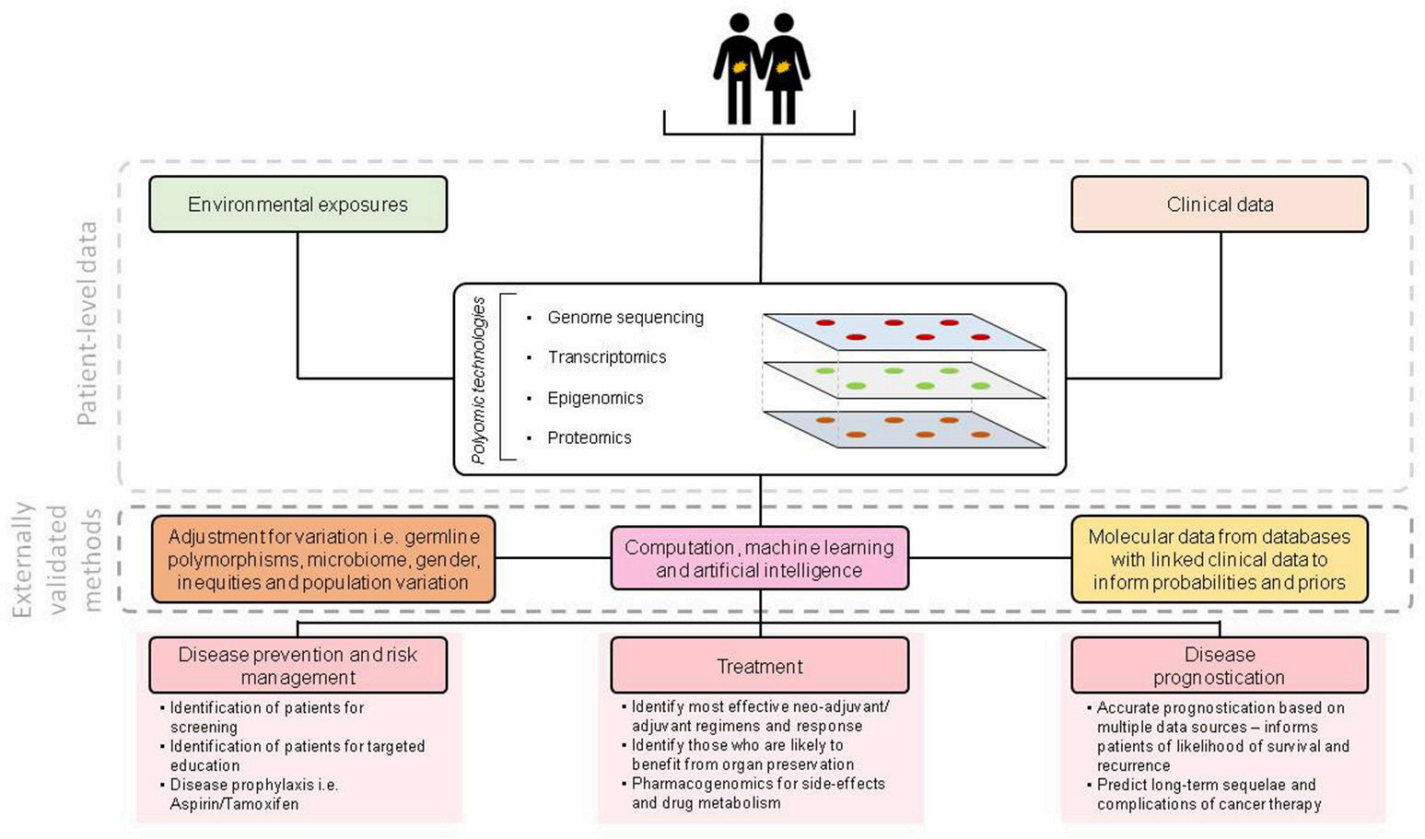
Overview of molecular technologies for enabling precision medicine.

Revolutionary polyomic (genomic, epigenomic, transcriptomic, proteomic, Figure 1) technologies have become established in clinical research and are increasingly commonplace in clinical practice in HICs. These technologies are set to revolutionise how research is performed and how therapies are selected—bringing precision medicine closer to reality than ever before. Indeed, the pharmaceutical industry are producing new agents designed to target specific molecular subtypes of disease. New prognostic modelling techniques, incorporating molecular data, clinical data and machine learning will provide more information to inform treatment choices for both patients and clinicians.

These evolving capabilities are set to transform outcomes, however, there is little consideration given to how these technologies can improve cancer outcomes across the world and applicability to LMICs. Despite these remarkable advances, most research and clinical trials are conducted on populations within HICs, thus limiting global generalisability. There is clear evidence that basic cellular processes vary across different human populations (3, 4). Despite this, only 3% of genome-wide association studies have been performed in Africans, without considering sequencing studies (5, 6). Evidence in HICs from studies comparing individuals from different ancestries has found that despite controlling for socioeconomic factors and other environmental exposures, there is still a large disparity in cancer incidence and outcomes that remains to be addressed (7, 8). To prevent advances in technologies creating even greater disparity in cancer care across the world, work must now be expanded to include low and middle-income settings.

## Cancer in low and middle-income settings

Across the spectrum of country development and geography, there are marked differences in the burden of cancer-related disease. Compared to well developed countries, LMICs have a higher age-standardised rate of gastric, oesophageal, bladder and liver cancers (Table 1). Although LMICs appear at present to have lower rates of lung, colorectal, pancreas and haematological malignancies, the 2016 Global Burden of Disease study has identified sustained rises between 1990 and 2016 of healthy years of life lost to these cancers (2).

**Table 1:** Relative increases in cancer burden by income setting.

**Cancer**	**HIC number of cases 1990**	**LMIC number of cases 1990**	**HIC number of cases 2016**	**LMIC number of cases 2016**	**Fold change HIC**	**Fold change LMIC**
Breast cancer	467198	70634	726622	190102	1.56	2.69
Tracheal, bronchus, and lung cancer	476710	72750	746752	159990	1.57	2.20
Stomach cancer	256111	98378	292833	136618	1.14	1.39
Colon and rectum cancer	477269	47737	792174	112741	1.66	2.36
Other neoplasms	96362	39052	247574	105289	2.57	2.70
Liver cancer	80650	46993	189298	91647	2.35	1.95
Prostate cancer	419216	25137	899317	74721	2.15	2.97
Pancreatic cancer	99603	18608	192036	39197	1.93	2.11
Bladder cancer	133992	14391	213500	34771	1.59	2.42
Kidney cancer	92384	9864	160805	25876	1.74	2.62
Uterine cancer	89318	12357	188007	25635	2.10	2.07
Malignant skin melanoma	83987	2293	211113	5763	2.51	2.51

*Global Burden of Disease estimates for cancer incidence (raw case number) in High SDI (HIC) and Low Middle Income (LMIC) countries in 1990 and 2016. Fold change in these is displayed in right hand columns. Considerable rises in cancer incidence in LMICs can be seen*.

**Figure 2:**
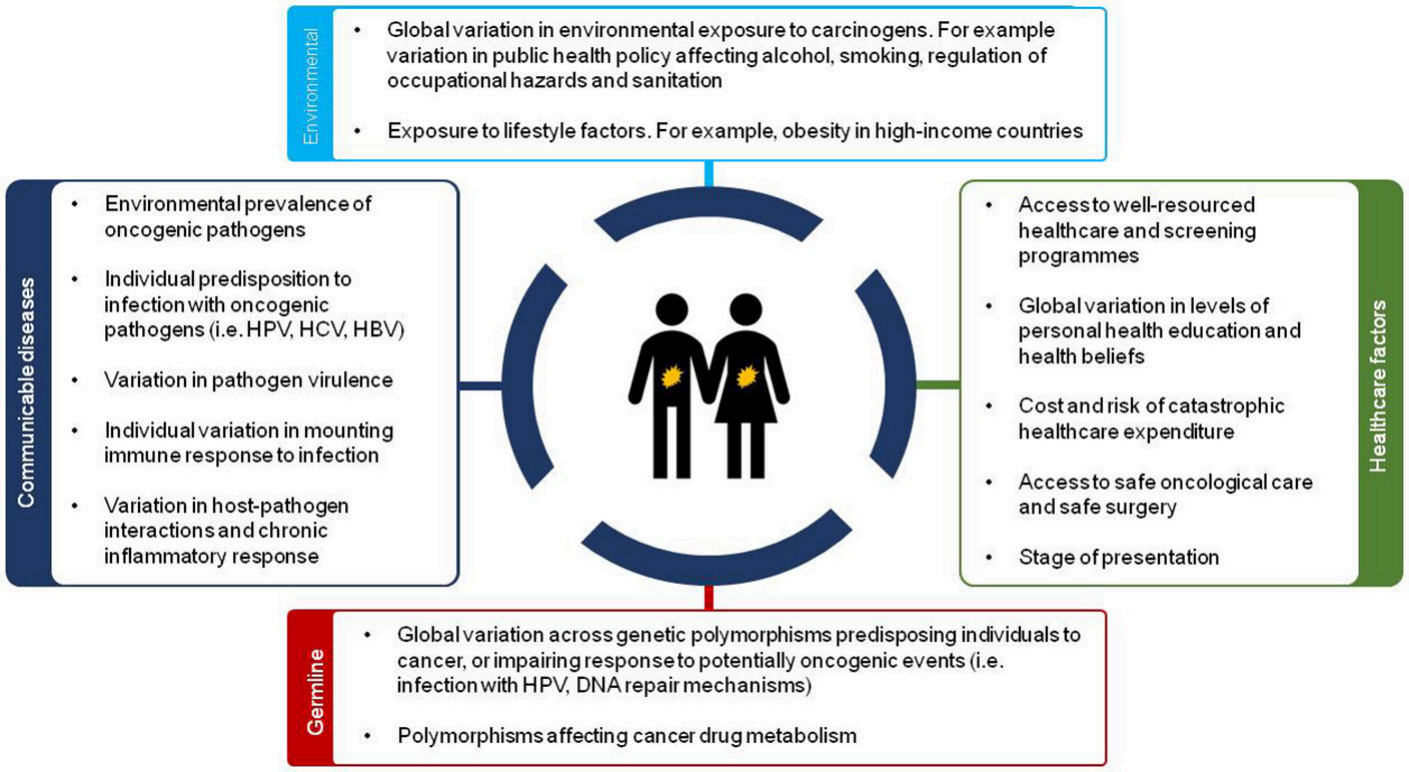
Overview of sources of disparity in future cancer care across the world.

This socio-economic and geographical variation in causes of cancer across the world has implications for both clinical practice and future research. This difference in disease profile is multifactorial (Figure 2) and includes environmental factors, infectious agents (i.e., hepatitis B (HBV), hepatitis C (HCV), Human Immunodeficiency Virus (HIV), and *Helicobacter pylori*), occupational exposures and other lifestyle factors (smoking, alcohol use) amongst others. In addition to considerable differences in these risk factors, lower levels of resources for healthcare and education result in patients presenting to healthcare facilities with advanced disease in less developed countries.

It is important to consider the context of cancer disease in LMICs can be somewhat different to developed settings and the implications this has. In this review we will explore how the molecular aetiology and epidemiology of cancer in less developed settings may differ and explore the impact this has for the future of clinical practice and research.

## Molecular divergence in cancer aetiology

Evidence suggests there is variation in both somatic, germline, and epigenetic alterations found across different human populations (3, 4). What is emerging suggests there are key genetic differences in some solid tumours when disease found in LMIC countries is compared to that in HICs (9–13).

In both African and middle-eastern countries, germline mutations in loci predisposing to breast, ovarian and colorectal cancer have been characterised in a small number of studies (14–19). In breast cancer for example, mutations in the BRCA1 and BRCA2 genes are more commonly found, with one Nigerian study identifying mutation rates of 7.9% for BRCA1 and 3.1% for BRCA2—far higher than in the cancer genome atlas (TCGA), where these are 1.3 and 1.5% respectively (16). Evidence from Tunisia and Morocco is concordant, with a higher rate of BRCA1/BRCA2 mutations, with some found to be novel, previously uncharacterised, of unknown clinical significance (14, 18, 20).

The same holds true for colorectal cancer, where there is little population-level data surrounding the prevalence of germline mutations in common cancer susceptibility genes. Germline mutations reported in the literature from African countries suggest that these variants are typically different from those found in HICs and the clinical significance of these mutations remains poorly characterised (7, 21–23).

Somatic and germline alterations across tumour types are not exclusively limited to low-middle income countries; racial disparity in the molecular composition of tumours also affects patients in HICs. Within the United States, for all cancers combined those of African-American ethnicity have been found to have mortality rates up to 25 percent higher than in Caucasian Americans (24). Several studies from TCGA and others have identified several key alterations in the somatic landscapes of tumours from African-Americans or Asian patients for renal, endometrial, breast, head and neck, colorectal, cervical and prostate cancers (25–29). Frequently, the differences in the mutational landscape of these tumours are in pivotal cancer driver genes, such as the von Hippel-Lindau (VHL) gene in clear cell renal cancer (26).

Furthermore, oncogenes are found to be differentially mutated in different ancestral groups, for example in endometrial cancer where PTEN mutations were found to predominate in those of Caucasian or Asian descent, whereas in African-Americans TP53 mutations are more common. These variations across ancestral groups extend to the nucleotide level, with mutational signatures having significantly divergent nucleotide signatures when compared to mutations found in Caucasian populations (27). Several initiatives are underway to address this, such as the American Association for Cancer Research (AACR) 2020 by 2020, which aims to sequence matched normal tissue and tumour for 2020 African-American cancer patients by 2020 (30). It is unclear whether the 2020 by 2020 initiative would utilise whole exome or whole genome sequencing. The latter would give a far richer pool of information on which to base further studies upon.

Globally, projects aimed at defining the human genome and cancer biology on a regional basis are beginning to emerge. For example, the International Cancer Genome Consortium (ICGC) is aimed at developing a comprehensive description of genomic, transcriptomic and epigenomic changes in 50 tumour types, with data from 16 countries already included (31). Recent publications have characterised the whole genomes of 560 breast cancers, demonstrating more than 90 mutated cancer genes were implicated in carcinogenesis, and that the mechanisms underlying most mutational signatures are presently unknown (32, 33). Unfortunately, LMICs (i.e., sub-Saharan Africa) are poorly represented within the project, as with many international genome collaborations, which limits conclusions and applicability on a truly global scale.

### Pharmacogenomics

Genetic polymorphisms affecting the metabolism of chemotherapy drugs may also be different across different ancestral groups. Differences in frequencies of functional genetic variants in key drug response and metabolism genes may significantly influence drug response differences in different populations (34–37).

Evidence from LMICs across the world is sparse, however, studies examining ethnic groups within HICs has identified substantial differences in treatment response and toxicities across ethnic groups. Understanding how these polymorphisms affect treatment response and side effects is important if precision medicine strategies are to be successfully implemented worldwide. These polymorphisms found across different ethnic populations can be beneficial or harmful. For example, African-Americans are more likely to have variants of the DPYD and TYMS genes which predispose this group to haematological toxicities with 5-fluorouracil as compared to Caucasian-Americans (38). Conversely, with 5-fluorouracil, Caucasian-Americans are more likely to suffer diarrhoea, nausea, vomiting, and mucositis when compared with their African-American counterparts. A similar picture is true in the metabolism of Doxorubicin, where African-Americans are more likely to suffer cardiotoxicities than Caucasian-Americans. Polymorphisms found in those of African ancestry may lead to life-threatening toxicities, such as neutropaenia. A fall in neutrophil counts following chemotherapy is more commonly found in patients of African-American and Asian descent when compared with Europeans (39). This may be due to a constitutionally lower neutrophil count (in the absence of cancer therapy), which has been associated with the presence of the Duffy antigen/receptor chemokine gene (DARC) rs2814778 SNP in a study examining 261 healthy volunteers (40). Several small studies examining the cytochrome P450, have identified polymorphisms across ethnic groups (41). Diversity in alleles of P450 is greatest across the African continent, compared to in Europe, and Asia. Where the CYP2B6, CYP2C8, CYP2D6, CYP2D6, CYP2D6, CYP3A5, and CYP3A5 have greatest diversity. Drugs associated with varied metabolism in the presence of polymorphisms affecting these genes include cyclophosphamide (CYP2B6^*^6), paclitaxel (CYP2C8^*^2) and 5HT_3_ receptor antagonists (CYP2D6). Despite this, the clinical implications these polymorphisms have for cancer therapy in the context of LMICs are poorly characterised. The polymorphisms found across these populations should be considered in the context of the healthcare infrastructure available. If patients in LMICs have similar diversity in polymorphisms associated with drug metabolism, then consideration must be given to the risk of exposing these patients to serious chemotherapy toxicities. Work is currently underway to try and identify polymorphisms associated with the metabolism of drugs found on the WHO's essential medicines list, beginning with HIV, which could be extended into cancer therapeutics and provide useful information to those administering treatments in LMICs (34, 42). Whole-genome precision medicine approaches to pharmacogenomics at the individual patient level are likely to be some way off, however, a precision medicine approach to public health could have significant advantages. In some ways, this population level consideration of the genetic diversity within a given population has started to occur. For example, in Ethiopia studies have revealed that a high proportion of the population are rapid codeine metabolisers due to CYP2D6 polymorphisms, leading to rapid conversion of codeine to morphine and subsequent overdose at therapeutic doses (100).

## Molecular diversity affecting communicable causes of cancer

Communicable diseases contribute toward a considerable proportion of the cancer burden in LMICs. These are potentially preventable cancers, with infectious agents commonly arising from poor sanitation, vertical transmission (mother to child), horizontal transmission (person to person) and a lack of safe healthcare practices (i.e., needlestick injuries and reused sharps). Examples include infectious agents such as Hepatitis B Virus (HBV), Hepatitis C Virus (HCV), Human Immunodeficiency Virus (HIV), human papilloma virus (HPV), and Epstein-Barr virus (EBV), which are well known for their oncogenic potential. Prevention programmes over the past 20 years have increasingly begun to recognise this, and vaccination programmes aimed at preventing hepatitis B have been shown to be effective in reducing the incidence of hepatocellular carcinoma. Similar can be seen for HPV vaccination, where programmes have begun to be rolled out in an increasing number of LMIC settings (43). Despite these initiatives, little is known as to the molecular landscape of these organisms and subsequent host-pathogen interactions.

### Hepatocellular carcinoma (HCC)

The effects of viral hepatitis on the development of hepatocellular carcinoma is well characterised in HICs. With global vaccination programmes aimed at preventing HBV underway, we may observe a decrease in HBV associated HCC. Despite this, HCC is multifactorial and infection with HCV or other causes of cirrhosis typically contribute to the risk of HCC development. The somatic landscape of HCC has been well characterised in American, European, Chinese and Japanese populations; however, evidence is lacking on disease found in LMICs (44, 45). The practicalities HCC poses to obtain tumour samples in LMICs are challenging, primarily owing to the risks associated with liver surgery and very late stages of presentation.

The distribution of HBV and HCV across the world has substantial variation and drive HCC formation in separate manners. HBV has a higher prevalence in LMICs and is responsible for the majority (%) of virus-induced hepatocellular carcinoma, compared with HCV (%) (46, 47). Dysregulation of key cell-cycle proteins, including cyclin dependent kinase 2 and 4, upregulation of the RAS/MAPK/ERK pathways and maintenance of upregulated canonical Wnt signalling, on the background of chronic inflammation are believed to initiate and drive HCV-related hepatocellular carcinoma (HCC) (48). HBV integrates within the host genome, initiating HCC through the promotion of genomic instability (49, 50).

Different genotypes of the Hepatitis C virus are known to lead to higher risk of HCC (51). Classically, the type 1b genotype has been associated with the highest risk of HCC formation. The global prevalence of HCV genotypes and variation worldwide has been studied in detail. Modelling studies demonstrate a high level of variation across the world in genotype, even within continents (46). Type 1 HCV predominates worldwide, however in central and west Africa, type 4 is more commonly found. Little data is available on why this variation exists and how this may reflect in disparity in HCC rates worldwide (46). In particular, study of genotype 4 and how this type mediates HCC formation in LMICs is missing. Host-pathogen interactions are known to play a crucial role in the clearance of these viruses and hence the subsequent risk of developing virus associated HCC. It is well known that patients of African ancestry have lower viral clearance rates than Caucasians (52). Several genome-wide association studies performed on patients of African descent have identified polymorphisms in alleles near class II Human Leukocyte Antigens on chromosome 6, the IL28B gene and other SNPs (53–56). This evidence draws upon a limited number of participants from LMICs and does not study the subsequent likelihood of HCC development.

The interaction between environmental and genetic factors may have a significant influence on the risk of HCC. The study of these interactions is limited in LMICs, however there are some examples of where this has been proven to be successful. Environmental exposures such as Aflatoxin B1 (found in certain grains and funghi), alcohol use, obesity, amongst others are known to contribute (57, 58). The interaction between Aflatoxin B1 and host genetics is particularly interesting: Aflatoxin B1 exposure is associated with HCC expressing more p53 mutations than in unexposed patients and may lead to greater genomic instability. Aflatoxin B1 has synergy with HBV, promoting HCC formation (59). Strategies to identify ways to abrogate DNA damage exerted by Aflatoxin B1 are under investigation and other environmental exposures are currently under study. Nevertheless, owing to little data on the molecular landscape of HCC in LMICs, it is unclear whether these research findings will have benefit for these patient groups. Similar epidemiological association studies have been done for other cancers, however, the data which provides the basis for disease models and translation to clinical practice in LMICs is lacking.

### Cervical cancer

Cervical cancer disproportionately affects women in LMICs, with the highest incidence found in sub-Saharan Africa (1, 60). Cervical cancer deaths are higher in LMICs, with 9 out of 10 deaths from cervical cancer worldwide occurring in LMIC settings. At present radiotherapy and surgery are the mainstays of cervical cancer management. The cause for this disparity in cancer incidence across the world is poorly understood and is multifactorial. Availability of radiotherapy in LMICs is also known to be extremely limited (61).

A proportion of cervical cancer cases are preventable, through early identification of dysplastic disease and immunisation against HPV. There are close connections with HIV too, with women who are HIV at a higher risk of developing cervical cancer. The prevalence of HPV is higher in women in LMICs than in HICs (62). There is a particularly high prevalence in Africa and Oceania, with higher exposure at a younger age. This in part accounts for the higher burden of cervical cancer in LMICs and the epidemiological burden of HPV associated cervical cancer is well described. In HIC populations genetic associations have been associated with the development of cervical cancer and are relatively well characterised (63, 64). Host-pathogen interactions between HPV and the immune system are also relatively well-characterised with respect to disease found in HICs. In HICs, genes such as TGF-β, those governing toll-like-receptors (TLRs), MHC genes and expression of cytokines, intricately linked with immune responses are associated with effective clearance of HPV (65–67). The somatic landscape of these tumours in LMICs is poorly characterised.

Evidence in LMICs suggests polymorphisms on the TYMS and RPS19 genes are associated with high-risk infection in Nigerian women, but overall in LMICs evidence is lacking on factors influencing effective HPV clearance (68). Several studies of women in HICs of African ancestry have identified that women of African-American descent despite having a similar prevalence of HPV, take longer to clear the virus (69). This delayed clearance, combined with known polymorphisms affecting genes responsible for immune response may go some way to explaining the higher rates of cervical cancer in LMICs (70). Further evidence is emerging that the distribution of HPV genotypes differs across separate geographical locations, with HPV 16 predominating worldwide, but HPV 58 and 31 more commonly found across Africa and East Asia. HPV 58 has been associated with increased risk of cervical cancer (71). Despite this work, there are scarce data from studies into virus genotype and genome-wide factors that may drive cervical cancer in LMICs.

## Barriers to global research in precision medicine

Genetic variation through germline polymorphisms and somatic mutations associated with cancers in LMIC populations, suggests there may be opportunity for implementing global strategies for more effective individualised treatment and better prognostication. In HICs, molecular testing is already being used to target therapies to specific alterations in tumours. Examples of this include the use of trastuzumab following HER2 testing in breast cancer, use of endocrine therapy in breast cancer and guiding EGFR targeted therapies in colorectal cancer using KRAS testing. In the case of colorectal cancer, both type and location of KRAS mutations are known to predict response to EGFR inhibition in colorectal cancer (72). There is some limited evidence to suggest EGFR/KRAS mutations occur in a similar pattern and distribution in LMICs as compared to HICs (22, 73, 74). However, in African-American populations within HICs, there is an increased frequency of mutations found in genes implicated in EGFR signalling, which may correspond with more aggressive disease (75, 76). Despite this, no clinical trials have aimed to include patients within these settings or sequence tumours to identify predictors of response. Building evidence to support precision medicine at a global level is important, both in terms of providing effective cancer therapies and being able to decide whether targeted therapies are cost effective in LMICs.

Building a sustainable workforce of clinicians, laboratory medicine and scientific leaders in this field is key. At present, in LMICs, implementing effective national programmes for precision cancer therapy and prevention following similar models to examples within HICs is unlikely to be feasible. A lack of trained laboratory medicine workforce, instruments, transportation, finances, and evidence to support the applicability of clinical response are all key factors (77–79). Access to pathology and laboratory medicine services in their current format is a major issue, with some LMICs having no workforce at all (77, 78). To support the implementation of precision medicine approaches to therapies, there is a pressing need to change this and ensure the emerging workforce have the skills to support the transition to precision medicine in these settings. Alongside the development of cancer therapies and research models, delivery of increasingly complex therapies requires major improvements in healthcare infrastructure and resource and it is optimistic to say that workforce development alone will enable this. Establishing infrastructure to support translational research in LMIC settings has challenges. The use of regional biobanks may provide a method to collect tissue samples now, for processing later, when infrastructure is in place, or even to demonstrate feasibility of systematic tissue collection—derisking investments that would otherwise have been spent on building an entire sample handling and sequencing pipeline. Biobanks, however, require large amounts of energy resources to ensure samples are frozen, with some biobanks using liquid nitrogen to freeze samples. Liquid nitrogen transport is difficult in HICs, let alone securing a reliable supply in LMICs. Electricity supplies in some LMIC locations is sporadic too, which would be required to operate freezer systems. Other preservative solutions could be used to preserve nucleic acids in tissues at room temperature as a stop-gap solution, or as a means of extending the time available to transport specimens to a central repository. Transporting biospecimens and tissue is subject to tight United Nations control, thus making international efforts more difficult for countries without existing expertise to contribute. Furthermore, times of epidemic and regional spread of disease has implications for whether it would be safe to transport biospecimens across the world. Further issues surrounding the logistics remain and policy makers should identify solutions to this as a priority.

### Global cancer trials

Conducting high-quality cancer trials is challenging in high-income settings and even more so in locations with little clinical trial infrastructure (80). Considerable methodological challenges exist around patient stratification by treatment response. Biomarker identification and sample handling must be robust and timely, together with the requirement for high levels of patient follow-up. In all countries, undertaking follow-up, and transporting biological specimens is challenging. These challenges are amplified in LMIC settings, with many countries lacking postal address systems, patient records and the infrastructure to process clinical specimens. Furthermore, a supply of clinical triallists is short in LMIC settings. Training and exchange programmes with HIC partners may help provide solutions in the sohrt term, however, long term infrastructure building must be given priority.

One example of where this is changing is Rwanda, where electronic patient record systems are being introduced (81). Integrating clinical systems into research in such settings would enable efficient research to be undertaken. In HICs, registry-based trials provide an efficient means of producing follow-up data and this approach could be emulated in LMICs where electronic records exist. A further consideration to any future trials of precision in LMIC settings is whether this can be continued after the trial concludes, should an intervention prove effective. For this reason, details on sequelae of interventions should be collected to ensure health-care systems can handle any treatment related harms. Non-governmental organisations such as the World Health Organisation and other non-governmental organisations (NGOs) often perform scoping studies in LMIC countries, but few cancer specific trials have been iniated. These NGOs should consider whether building research infrastructure in medicine is a sufficient priority to enable tailored solutions to be led by LMIC investigators independently.

In other areas of medicine (such as malaria, HCV and HIV) have successfully delivered clinical trials that integrate molecular or genotypic testing to enable molecular determinants of disease response to be identified in LMICs (82). Malaria is a good example, where trials often collect data on genotypes and information on microsatellites of malaria parasites from DNA isolated from blood films or spots. The markers used can be tested in a polymerase chain reaction (PCR) assay by research staff at that centre. Malaria trials have used these to investigate markers of treatment resistance and response (83, 84). Similarly, for trials in antiretroviral therapy for HIV and HCV, there are several studies which utilise commercial molecular testing kits at the centre level for identifying genotypes found in disease (85). However, this approach is challenging to adopt and thought must be given to the sustainability of testing when it is attempted to adopt trial findings into routine clinical practice.

### Capacity building for precision medicine

The most notable effort currently underway in LMICs to build evidence and crucially capacity for genomic sequencing is the Human Heredity and Health in Africa initiative (H3Africa) (86). This project is a collaboration of African clinicians, scientists and bioinformaticians who conduct large-scale sequencing and genetic association studies. So far, they have largely focussed on communicable disease such as trypanosomiasis, stroke and other neurological diseases affecting patients across the continent. Relevant to the field of oncology, some work has been undertaken into HPV infection in Nigeria (68), with the women included in this study demonstrating similar genetic susceptibility to infection as other populations. This group are set to expand into the field of breast cancer, which will provide useful data to study the genomic epidemiology of the disease on the African continent. The MRC Centre for Genomics and Global Health in the Gambia has also conducted successful genome wide and sequencing techniques in other disease areas, including in *P. falciparum* (87, 88).

There have been several success stories in LMICs, with some African countries delivering exciting genetic epidemiology studies. The Nigerian Breast Cancer Study, led by the University of Idaban has produced several large studies, underpinning the understanding of breast cancer genetics in Nigerian women (89). Through collaborative support from the University of Chicago, this group has gone on to publish multiple genetic epidemiology studies, and have even attempted randomised clinical trials (90). Building partnerships between institutions with experience in polyomics may help foster knowledge exchange and promote the implementation of best practice.

### Local lead and oversight of research projects

Any solution must be led and maintained locally, rather than researchers from HICs taking data from local populations. It must also maintain practicality and clinical relevance to the local patient population. The H3Africa initiative has recently published guidelines for researchers wishing to do research in the African continent which are aimed at empowering local scientists and ensuring benefit for African patients (5). Developing home-grown expertise in polyomic technologies in LMICs would also have economic benefits in addition to building the global laboratory medicine workforce of the future. In fact, LMICs may be at some advantage in building competitive platforms for precision medicine as they can adopt new technologies without having to make mistakes or go through the intermediate steps of technological evolution that have been observed in high-income settings.

### Disruptive near-future technologies

**Figure 3:**
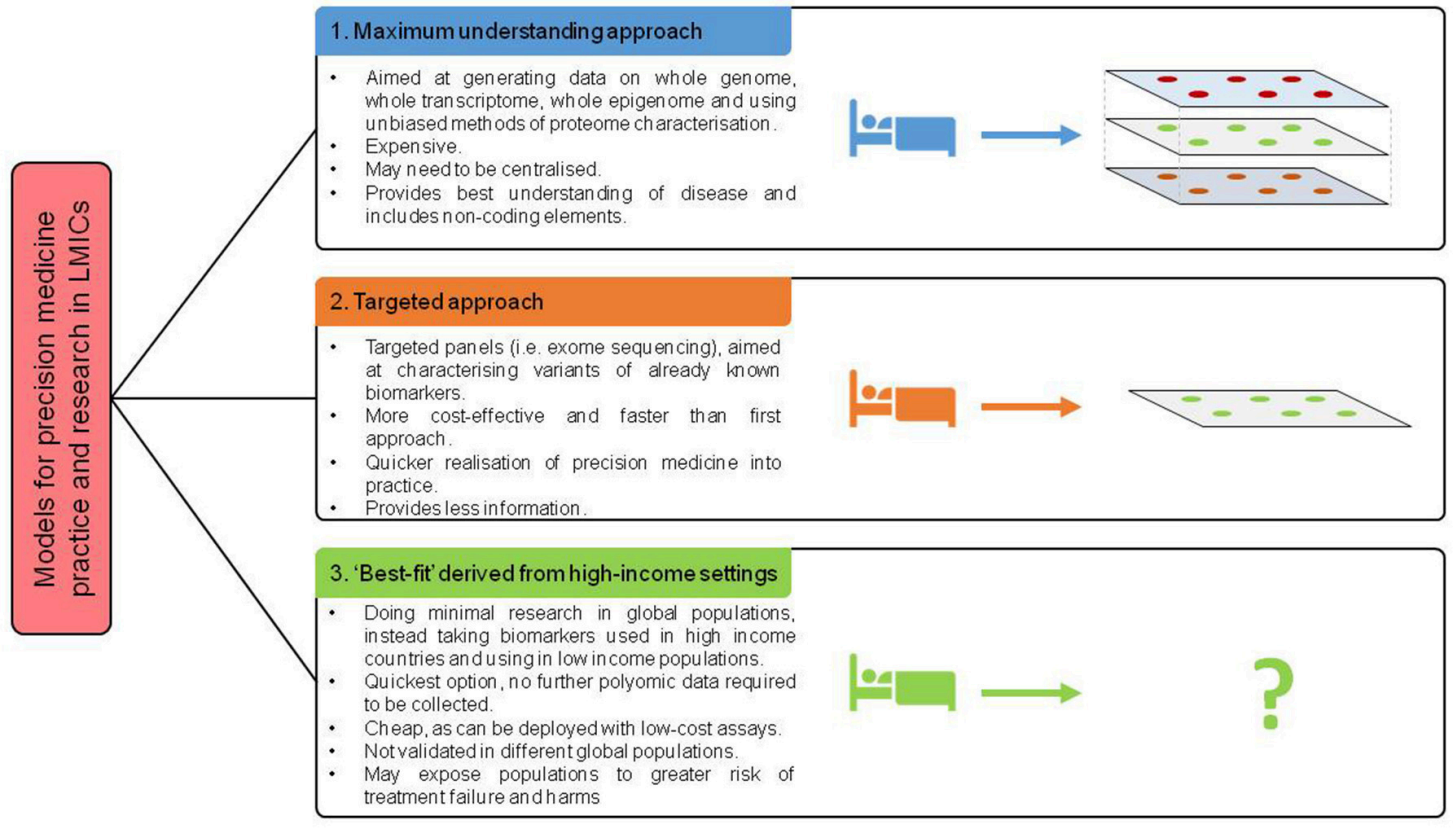
Proposed models of future deployment of precision medicine across world.

New technologies may provide innovative solutions to some of the barriers to precision medicine. Genome sequencing over the past 15 years has fallen by 100,000-fold, yet with the cost of sequencing an entire human genome around the region of $1000, this is still prohibitively expensive for patients and healthcare systems (91). Furthermore, the sample handling and analysis pipelines required to support genome sequencing is logistically challenging. Establishing infrastructure and identifying expert staff to deal with sequencing data and ensure appropriate quality control is a large barrier. Large quantities of computational resource are required and may work as a centralised resource across, if shared across multiple LMICs. Whilst a centralised model may be a more cost and resource effective model, other challenges, including specimen transportation, and political stability could pose barriers to such a model. Furthermore, models of research and practice must be sensitive to resources of countries involved (Figure 3). Examples such as nanopore sequencing devices may provide an answer to these logistical challenges, with handheld and smartphone based sequencing devices available which reduce requirements for library preparation (92, 93). This has the potential to enable targeted sequencing of patients, at a far lower cost. With other multiplexing approaches, including developments in nucleotide barcoding, many samples could be rapidly processed at a very low cost.

Targeted sequencing and mutation detection assays (PCR or ligation based methods, Table 2) offer a good alternative, but require that there is data to support that these assays bring clinical benefit, often derived from sequencing data. New, more portable approaches to molecular testing are being developed, which may be useful in resource-limited settings. Paper-based oligonucleotide based ligation assays, originally developed to detect HIV genotypes and monitor resistance, could be adapted for use in cancer therapy (94, 95). Paper-based immunochromatography assays, popularised for detection of HIV, could also be adapted for the detection of cancer specific ligands at the point of care. Nevertheless, these approaches lack the ability to resolve molecular alterations at the nucleotide level and rely on these already being known. The potential impact for these assays is great, particularly in the context of cancer diagnosis in limited resource settings, where other common diagnostic tests such as endoscopy or imaging maybe unavailable.

**Table 2:** Possible enabling technologies for global precision medicine.

**Technology**	**Pros**	**Cons**	**Barriers to implementation of technologies to LMIC settings**
Whole-genome sequencing (Sequencing-by-synthesis or ion semiconductor sequencing)	High-throughput and high-speed Well developed technology Sequencing pipelines can be developed with ‘off the shelf' solutions Large body of global expertise Provides detailed, pangenome information Deep sequencing would provide unparalleled information on novel variants (including non-coding)	High cost Likely to require centralisation of expertise due to lack of infrastructure currently Sample preparation and library generation required Sample logistics may be difficult from the perspective of clinical care and transporting sample to centralised facilities Short reads	Current lack of computational infrastructure and expertise in LMICs Likely to require international cooperation - could be sensitive to political instability Sample pipelines would require careful planning and implementation High cost
Exome-sequencing (Sequencing-by-synthesis or ion semiconductor sequencing)	Lower cost than whole-genome Still captures information on important genes Typically faster than whole genome sequencing Could be performed using a more regional model of delivery Cheaper sequencing instruments to deliver same depth as whole genome More clinically relevant as same infrastructure could deliver targeted clinical panels	As above for whole genome sequencing Still relatively high cost Offers less coverage and no coverage of non-coding elements	As above for whole genome sequencing
Direct sequencing (Nanopore sequencing)	Highly transportable Minaturised versions available that require less computational infrastructure than other sequencing approaches Lower cost than other sequencing instruments Less sample and library preparation Can be used to directly sequence other nucleic acids and proteins Easily expandable Very long read lengths	Currently limited to targeted sequencing studies in humans Currently marginally lower accuracy than established semi-conductor and sequencing by synthesis approaches Limited depth at present in humans versus other approaches Clinically approved devices not yet available	Would require international collaboration on how data is pooled together and standard operating procedures to ensure quality control if many users and devices used in a distributed model Current lack of computational infrastructure and expertise in LMICs
Other targeted panels (i.e. microarray)	Lower cost than sequencing High throughput Less computationally intensive Other applications i.e. cytogenetics Global expertise readily available Cheap instrumentation	Biased detection methods Sequencing becoming increasingly more popular Lower dynamic range for detection than sequencing methods Cannot detect novel transcripts	Would require international collaboration on how data is pooled together and standard operating procedures to ensure quality control if many users and devices used in a distributed model Technology may be outdated and superceded by sequencing Current lack of computational infrastructure and expertise in LMICs
Oligonucleotide ligation assays/ Polymerase chain reaction	Very cheap Can be paper-based Transportable Limited scientific skills required Easily mass-produced	Can only detect known variants in a very simple fashion Not quantitative Requires substantial development Unclear how may be used with heterogenous samples i.e. solid tumour Data not easily stored in electronic format	Would require sequencing or array studies to validate targets of assays prior to deployment

Promising developments are on the horizon for the diagnosis and monitoring of disease, such as liquid biopsy and measurement of circulating nucleic acids (circulating tumour DNA/ RNA) (96). At present, these technologies rely of measurement of small quantities of nucleic acids, circulating tumour cells or other targets in peripheral blood via sequencing and subsequent computational processing (97). Although a reliable and universal test is yet to be developed based on this methodology, the development of such a test has the potential to improve early diagnosis and reduce reliance on other expensive methods of diagnostic testing in LMICs. However, given the lack of workforce, infrastructure and expertise available in LMICs at present, new technologies may further exacerbate global inequalities. Similarly, for other biomarkers of cancer which could be utilised as substrates to bind ligands to deliver cancer therapies with (such as fluorescence imaging (98), chemotherapeutics, or radiotherapeutics), there is little data to support whether what works in HICs can be translated straight into LMICs safely. Indeed, at present there is very little data that underpins the patient pathway or outcomes after cancer treatment in LMICs.

Collaborative approaches are crucial to ensuring future success (80, 99). Several partnerships between HICs and LMICs have begun to collect prospective, high quality evidence and establish clinical research networks. One such example is the GlobalSurg collaborative, a network of over 5,000 surgeons throughout the world who deliver large multicentre prospective studies (100). Recently, this network has established several trials units in LMIC settings that will deliver cancer trials and test whether new devices being concurrently developed in HICs can be utilised in LMICs. Building sustainable capacity concurrently with new developments will enable local economies to thrive and patients in LMICs to receive cutting edge care. Working with LMIC partners to facilitate global translation should be a priority when developing potentially disruptive technologies for both researchers and industry.

## Summary

Funders, scientists, genome consortia, scientific journals and policy makers have an important role to play in a drive to ensure cancer research is generalisable across the world and will benefit patients in LMICs. Many major journals now mandate that polyomic data is deposited in databases for future use by the scientific community and other interested partners. This has been highly successful, with databases such as RNAcentral containing 13 million sequences and the ICGC Project available to the worldwide scientific community (31, 101). In addition, collaboratives such as the 1,000 genome project, containing 2,500 human genomes and representing 26 populations, demonstrate the growing potential to sequence genomes on a global scale (102). However, most data currently deposited is from HICs and more work is required to increase the availability of data from LMICs. Funders have begun to recognise this, and recent discussions have begun to focus on building precision medicine at a population level in LMICs (5, 103). At present, this initial funding is focussed on precision medicine for communicable disease, despite the rising burden of non-communicable disease.

Precision medicine clearly offers numerous advantages for patients and recent efforts to characterise the landscape of cancers using polyomic technologies is changing research and practice. There is, however, a lack of evidence, data and clear strategy on how this will be used to benefit patients across the world, particularly in LMICs. Emerging evidence suggests there are differences at the molecular level between cancer in HICs and that found in LMICs. To ensure inequity in cancer care between high and LMIC settings is not worsened, steps must be taken to improve the mechanistic understanding of cancer at a global level.

## Summary points

- There is known variation in basic cellular processes such as DNA methylation, epigenomic alterations and frequencies of polymorphisms across different human populations. These are likely to affect cancer risk, disease behaviour and treatment response.
- LMIC populations are under-represented in large genome wide association studies and sequencing studies. In an era where genomic technologies are driving drug development and targeted therapies, this may result in global inequities for cancer therapy.
- To prevent cancer inequities worsening further, funders, researchers and scientists should aim to include patients from LMICs in international studies. This would ensure that emerging consensus molecular subtypes are representative of disease worldwide.
- New technologies present exciting opportunities to improve cancer care and the representation of LMIC countries in cancer research. Further work should be done to ensure LMIC representation and identify novel ways of implementing cost-effective approaches to precision medicine or precision public health within LMIC settings.

## Author contributions

All authors listed have made a substantial, direct and intellectual contribution to the work, and approved it for publication.

### Conflict of interest statement

The authors declare that the research was conducted in the absence of any commercial or financial relationships that could be construed as a potential conflict of interest.
